# Pregnancy in Chronic Kidney Disease: Need for Higher Awareness. A Pragmatic Review Focused on What Could Be Improved in the Different CKD Stages and Phases

**DOI:** 10.3390/jcm7110415

**Published:** 2018-11-05

**Authors:** Giorgina B. Piccoli, Elena Zakharova, Rossella Attini, Margarita Ibarra Hernandez, Alejandra Orozco Guillien, Mona Alrukhaimi, Zhi-Hong Liu, Gloria Ashuntantang, Bianca Covella, Gianfranca Cabiddu, Philip Kam Tao Li, Guillermo Garcia-Garcia, Adeera Levin

**Affiliations:** 1 Department of Clinical and Biological Sciences, University of Torino, 10100 Torino, Italy; 2 Néphrologie, Centre Hospitalier Le Mans, 72000 Le Mans, France; biancacovella@gmail.com; 3 Nephrology, Moscow City Hospital n.a. S.P. Botkin, 101000 Moscow, Russia; helena.zakharova@gmail.com; 4 Nephrology, Moscow State University of Medicine and Dentistry, 101000 Moscow, Russia; 5 Nephrology, Russian Medical Academy of Continuous Professional Education, 101000 Moscow, Russia; 6 Obstetrics, Department of Surgery, University of Torino, 10100 Torino, Italy; rossella.attini@gmail.com; 7 Nephrology Service, Hospital Civil de Guadalajara “Fray Antonio Alcalde”, University of Guadalajara Health Sciences Center, Guadalajara, Jal 44100, Mexico; maribaher@yahoo.es (M.I.-H.); ggarcia1952@gmail.com (G.G.-G.); 8 Instituto Nacional de Perinatologia, Mexico D.F. 01020, Mexico; ale_gaba@hotmail.com; 9 Department of Medicine, Dubai Medical College, P.O. Box 20170, Dubai, UAE; mona_539@yahoo.co.uk; 10 National Clinical Research Center of Kidney Diseases, Jinling Hospital, Nanjing University School of Medicine, Nanjing 210000, China; zhihong--liu@hotmail.com; 11 Yaounde General Hospital & Faculty of Medicine and Biomedical Sciences, University of Yaounde I, P.O. Box 337, Yaounde, Cameroon; maglo09@hotmail.com; 12 Nefrologia Ospedale Brotzu, 09100 Cagliari, Italy; gianfranca.cabiddu@tin.it; 13 Prince of Wales Hospital, Department of Medicine and Therapeutics, Chinese University of Hong Kong, Hong Kong; philipli@cuhk.edu.hk; 14 Department of Medicine, Division of Nephrology, University of British Columbia, Vancouver, BC V6T 1Z4, Canada; alevin@providencehealth.bc.ca

**Keywords:** chronic kidney disease (CKD), dialysis, kidney transplantation, pregnancy, pregnancy complications

## Abstract

Pregnancy is possible in all phases of chronic kidney disease (CKD), but its management may be difficult and the outcomes are not the same as in the overall population. The prevalence of CKD in pregnancy is estimated at about 3%, as high as that of pre-eclampsia (PE), a better-acknowledged risk for adverse pregnancy outcomes. When CKD is known, pregnancy should be considered as high risk and followed accordingly; furthermore, since CKD is often asymptomatic, pregnant women should be screened for the presence of CKD, allowing better management of pregnancy, and timely treatment after pregnancy. The differential diagnosis between CKD and PE is sometimes difficult, but making it may be important for pregnancy management. Pregnancy is possible, even if at high risk for complications, including preterm delivery and intrauterine growth restriction, superimposed PE, and pregnancy-induced hypertension. Results in all phases are strictly dependent upon the socio-sanitary system and the availability of renal and obstetric care and, especially for preterm children, of intensive care units. Women on dialysis should be aware of the possibility of conceiving and having a successful pregnancy, and intensive dialysis (up to daily, long-hours dialysis) is the clinical choice allowing the best results. Such a choice may, however, need adaptation where access to dialysis is limited or distances are prohibitive. After kidney transplantation, pregnancies should be followed up with great attention, to minimize the risks for mother, child, and for the graft. A research agenda supporting international comparisons is highly needed to ameliorate or provide knowledge on specific kidney diseases and to develop context-adapted treatment strategies to improve pregnancy outcomes in CKD women.

## 1. Introduction

Chronic kidney disease (CKD) is a well-acknowledged risk factor for adverse pregnancy outcomes [1,2,3,4,5,6,7]. The literature on this issue is rapidly accumulating and the term “obstetric nephrology” has been proposed to identify this important clinical and research field [8].

However, awareness of the importance of identifying CKD in pregnancy is still insufficient and the experience is mainly limited to some large, but still few, referral centers [3,9,10,11,12].

This narrative review, in association with a review on pregnancy and acute kidney injury (p-AKI), has been based on the theme of the World Kidney Day 2018, which highlighted women’s health and kidney disease [13]. Importantly, the focus of this narrative is on what could be done to improve CKD care throughout and after pregnancy.

The review follows the classic, even if not necessarily sequential, phases of renal diseases: chronic kidney disease, dialysis, and transplantation, and focuses on the knowledge gaps, on the delay of application of what is known into the clinical practice, and on the potential interventions that could improve the care of mother and child during and after pregnancy.

## 2. Chronic Kidney Disease

### 2.1. State of the Art: What We Know on the CKD-Pregnancy Relationship: CKD Stages

Kidney function is of crucial importance in healthy pregnancy [8].

Several changes in kidney function occur in the pregnant woman, affecting the vascular, glomerular, and tubular components, ultimately resulting in increased renal clearances and “physiological” proteinuria, decrease in blood pressure, and expansion of the intravascular volume [8,14,15,16,17].

The kidney is the target and the central player in the hypertensive disorders of pregnancy, an umbrella term that gathers the most common pregnancy-induced disorders: isolated hypertension (usually identified by the acronym PIH: pregnancy induced hypertension), pre-eclampsia (PE), in which hypertension is usually associated with proteinuria and may be associated with acute and transient reduction of the kidney function (now considered a hallmark of the PE syndrome, even in the absence of proteinuria), HELLP syndrome, an acronym for haemolysis, elevated liver enzymes, low platelets, a severe, occasionally life-threatening, endothelial disorder [8,14,15,16,17,18,19,20]. Isolated proteinuria may also transiently appear in pregnancy and is usually indicated as “pregnancy-induced proteinuria”. Proteinuria may precede PE, but even when isolated, it heralds a risk of adverse pregnancy outcomes, including growth restriction and preterm delivery; the differential diagnosis between pregnancy-induced and pre-existent proteinuria may not be easy [20,21,22,23].

Due to the central role of the kidney, target, and actor in the pathogenesis of the hypertensive disorders of pregnancy, it is not surprising that a reduction of the kidney function may affect pregnancy outcomes [1,2,3,4,5,6,7,8,9,24]. What may be surprising is that kidney diseases are associated with a significant increase in the risk of adverse pregnancy outcomes even in the absence of kidney function reduction [4,5,25,26,27,28,29,30].

In this regard, interesting insights come from the analysis of pregnancy after kidney donation, which shows that this condition of “healthy” reduction of the kidney parenchyma is associated with a higher risk of pre-eclampsia and hypertensive disorders of pregnancy [25,26,31,32,33].

Overall, the risks of adverse pregnancy outcomes increase from CKD stage 1 to CKD stage 5, and are further increased in diabetic nephropathy and in systemic autoimmune diseases, such as systemic lupus erythematosus (SLE) [1,2,3,4,5,6,7,8,9,34,35,36,37,38,39,40].

In each CKD stage, hypertension and proteinuria are important modulators of the entity of pregnancy-related risks; however, the specific role of each element (kidney disease, stage, hypertension, and proteinuria) is not fully known, thus limiting the information available for counseling [41,42,43,44] (Table 1). Furthermore, perinatal outcomes depend also upon the setting of care, an issue that has to be taken into account both in interpreting results and in planning treatment strategies.

As it will be further discussed, our knowledge on the specific risks linked with the different kidney diseases is limited; overall, we know more about glomerular diseases, and the most common ones, such as IgA nephropathy, are extensively studied; conversely, the specific risks associated with interstitial nephropathies or polycystic kidney disease (ADPKD) are not fully appreciated [7,9,45,46,47,48,49,50,51].

Table 2 resumes the main pregnancy-related risks in CKD patients: overall, malformations are not increased with respect to the overall population (out of the context of inherited diseases and of diabetic nephropathy); maternal death is exceptional, at least in highly resourced countries.

**Figure 1:**
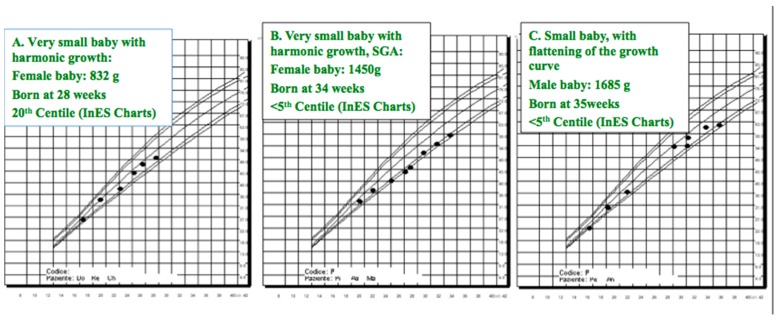
The different “small babies”: Growth curve of small, small for gestational age (SGA), intrauterine growth restricted (IUGR) babies (Y axis: weight and reference curves; X axis: gestational weeks). Legend: (**A**) is a very small, early preterm normal for gestational age child; (**B**) is a very small, early preterm, SGA but harmoniously grown, preterm child (mother and father also of small body size); child (**C**) is a small, SGA, preterm child with a flattening of the growth curve. Although (**B**,**C**) are identified also as IUGR, flattening of the growth curve may have a different (unfavorable) meaning as for life-long complications. (Courtesy of R. Attini and P. Gaiotti).

Conversely, the incidence of preterm delivery and of small babies (Figure 1) is increased already in stage 1 CKD, with respect to the overall population, and rises along with the increase of CKD stages [1,2,3,4,5,6,7,24,27,28]. Likewise, the effect of pregnancy on CKD progression is debated, also on account of the different study designs, obstetric policies, and duration of follow-up of CKD women after pregnancy. Overall, short- and long-term decrease in the kidney function is exceptional in the first CKD stages, but rapid decrease of the kidney function may be an issue in late CKD stages [1,2,3,4,5,6,7].

Once more, studies are heterogeneous and evidence is limited; as a consequence, entity of the risk of CKD progression has been variously estimated (Table 2).

The information on the outcomes of pregnancy in the last CKD phases is still scant. Fertility is usually reported as reduced in the last CKD stages, but it is possible that the attitude of discouraging pregnancy in advanced CKD has induced a selection bias [52,53].

### 2.2. A Particular Case: Systemic Immunologic Diseases

Kidney disease in SLE is a critical concern for pregnancy. Kidney involvement includes lupus nephritis (LN), characterized by glomerular damage and interstitial and vascular lesions. Beyond risk factors associated with CKD (proteinuria, hypertension, and impaired kidney function, Table 1 and Table 2), SLE is associated with a specific increase in miscarriages and perinatal death. All risks are higher in active SLE, and adverse outcomes are associated in particular with LN class 3 and 4, history of renal flares, longer disease, hypocomplementemia, presence of antiphospholipid antibodies (aPL), and antiphospholipid syndrome (APS) [5,6,7,54,55].

Conversely, pregnancy carries a risk of SLE flares: high estrogen level may act as triggers, by mediating transcription activity of the intracellular estrogen receptors and interacting with regulatory T cells, key modulators of maternal–foetal tolerance [56]. Upregulation of IFN-α may also play a role in SLE and LN: this cytokine, highly expressed by the placenta, contributes to placentation and increases susceptibility to SLE [57,58,59]. Low C3 and high anti-DNA antibodies predict renal flares, whereas high anti-C1q antibodies and low C4 predict early flares. 

Pregnancy complications, along with vascular thrombosis, are the main clinical criteria for antiphospholipid syndrome, which may be isolated (primary forms) or associated to SLE or other autoimmune disorders. The disease directly affects placental vasculature, ultimately resulting in placental dysfunction: antiphospholipid antibodies affect the cytotrophoblast via thrombosis, inflammation, apoptosis, and immunomodulatory impairment; direct damage of endometrial cells has also been described [60,61]. APS-related complications in pregnancy include the whole list of pregnancy complications: recurrent miscarriage, preterm delivery, IUGR, stillbirth, fetal distress, fetal or neonatal thrombosis, PE, eclampsia, HELLP syndrome, arterial or venous thrombosis, and placental insufficiency. High titers and triple positivity for aPL (usually defined as positivity for LLAC and for anti-cardiolipin (aCL) and anti-β2GPI antibodies of the same isotype by the same method) are associated with mother and foetal complications, including miscarriage [60,61,62,63,64,65,66,67,68,69]. A particularly severe syndrome, named catastrophic APS (CAPS), combines all these damages into multiorgan failure [60,61,62,63,64,65,66,67,68]. The occurrence of HELLP syndrome in a patient with APS should raise the suspicion of CAPS, and defines a permanent risk for further pregnancies [69].

The link between pregnancy-induced thrombocytopenia or endothelial damage and other causes, including thrombotic thrombocytopenic purpura (TTP) and hemolytic uremic syndrome (HUS), now often referred to as thrombotic microangiopathies (TMA), is not fully clear; the diseases may overlap and the differential diagnosis may become evident only after pregnancy (Table 3) [70].

Among other common autoimmune diseases, rheumatoid arthritis (RA) mostly affects women of perimenopausal age; however, its incidence is non-negligible in younger patients (8.7 per 100,000 per year at age 18–34 and 36.2 per 100,000 at age 35–44). CKD in RA may result from RA-associated glomerulonephritis, chronic inflammation, comorbidities, nephrotoxic antirheumatic drugs, and amyloidosis [71,72,73,74,75,76,77,78]. Specific pregnancy risks linked to RA are not clearly defined, even if an increase in preterm delivery and low birth weight has been reported (Table 1 and Table 2). Interestingly, RA patients may experience remission during pregnancy, presumably due to changes in sex hormones profiles [75,76,77,78,79,80]. Similar considerations may apply to systemic sclerosis (SS), a severe immunologic disease mostly affecting women of postmenopausal age, but present also in a younger population, where it carries a risk for prematurity and IUGR and SGA babies. Conversely, SS flares are rare but may be life-threatening in pregnancy [81,82,83,84].

### 2.3. What is Missing, and What We Still Need to Know

Indirect estimates of the prevalence of CKD in pregnancy come from the assumption that fertility is not affected in early CKD and that the prevalence of early kidney disease is the same as in the overall population [1,85]. Based upon this assumption, it has been suggested that early-stage CKD is present in 3:100 pregnancies and later-stage CKD in 1:750 pregnancies [1,85]. Interestingly, the reported prevalence of CKD is strikingly similar to that of PE, much better acknowledged as an important risk in pregnancy [1,85,86]. Furthermore, the estimation of the prevalence of CKD in pregnancy, in the classic paper by Williams and Davison, is based upon an evaluation performed in western, highly resourced countries, and may be underestimated in medium- and low-income countries, in which the prevalence of CKD is usually higher [1,85,86,87,88,89,90,91]

Assessing the actual prevalence of CKD in pregnancy should therefore be a health care priority, also since pregnancy may represent an underutilised, but highly valuable, occasion for the diagnosis of CKD, in particular in disadvantaged populations in which the opportunities for early CKD diagnosis are few [90,91,92,93,94]. In fact, pregnancy is often the first occasion in which an apparently healthy woman undergoes a clinical and laboratory evaluation, which has the potential to timely reveal the presence of underlying CKD. However, the only assessment systematically done and regarding the kidney is the urinalysis, which is usually interpreted only with regard to the presence of proteinuria, or of urinary tract infection [95,96,97].

Simple and inexpensive measures, such as including the assessment of serum creatinine in the routine pregnancy tests, could improve early diagnosis of kidney diseases, in particular if the physiological decrease of serum creatinine levels, due to physiological changes in pregnancy, is acknowledged. In this regard, there is also a need to determine reference values for creatinine during pregnancy, by race and pregnancy trimester. Addition of serum creatinine will not solve all problems, but may allow identifying at least cases with kidney function impairment. Indeed, many kidney diseases are fully asymptomatic; not all kidney diseases manifest with hypertension and proteinuria, the symptoms on which attention is more focused; haematuria or electrolyte derangements may be overlooked in pregnancy and there is a consistent overlap of signs and symptoms between PE and CKD in pregnancy [98,99,100,101]. These diagnostic challenges are summarised in Table 4.

Furthermore, signs and symptoms of CKD and PE may overlap, and there is a need for establishing common lines for systematically considering the differential diagnosis between CKD and PE in pregnancy; while a combination of Doppler flows and the biomarkers employed in the assessment of PE may support diagnosis, large prospective studies are needed to refine the procedures and validate their use [101,102,103].

## 3. Dialysis

### 3.1. The State of the Art

Fertility is reduced in end-stage kidney disease; Australian and European data suggest that pregnancy occurs ten times less frequently in patients with kidney transplantation than in the general population and that pregnancy occurs ten times less frequently in dialysis patients compared with kidney transplant patients. In other words, a woman on dialysis has a probability of having a baby that is 1% of the overall population [104,105,106].

While the first sporadic cases of successful pregnancy on dialysis were described in the seventies, it was only in the new millennium that pregnancy on dialysis became an acknowledged clinical possibility [104,105,106,107,108,109,110]. So far, more than 1000 pregnancies have been reported in dialysis patients, with an increasing trend worldwide [109].

The most important advance in this field has been the demonstration of a strict relationship between the intensity (frequency and duration) of the dialysis sessions and pregnancy results, thus leading to intensify dialysis up to daily, favoring also long-hours treatment as compared to standard schedules [107,108,109,110].

The improvement in results recorded with daily, extended-hours dialysis not only allowed a more permissive attitude towards pregnancy in dialysis, but is also leading to a more positive attitude towards pregnancy in advanced CKD, often previously discouraged for the fear of needing to start dialysis during gestation [107,108,109,110,111,112,113,114]. 

While the “best” (if any) dialysis prescription is still not agreed upon, some common lines are emerging, specifically from the Canadian experience: in patients without residual renal function, at least 36 h of dialysis per week should be prescribed, and daily frequency should be chosen; weight loss during the session should be very slow, hypotension should be avoided, and hypertension not hypercorrected; heparin anticoagulation is safe (more experience with unfractioned heparin); biocompatible membranes should be used; multivitamin supplementation and high-protein diets should be prescribed to compensate for intradialytic nutrient loss; phosphate supplements may also be needed. Low blood and dialysate flows should allow a “soft” dialysis; Kt/V is not the ideal marker of dialysis efficiency and the target is set at “near normal” predialysis urea (after the day break) (Table 4) [10,109,111].

In settings where dialysis is available without restrictions, pregnancy in dialysis is becoming more common, though still a rare occurrence, underlining the importance of a network of care for sharing opinions, gathering data, and optimizing results [110,114].

Conversely, in poorly resourced countries, pregnancy is a common precipitating event of severe CKD, often not previously diagnosed; furthermore, in these settings, p-AKI is more common and may not be reversible in 5–30% of the cases, further underlining the complex link between PE, p-AKI, and CKD in pregnancy [115,116,117,118].

### 3.2. What Is Missing and Could Be Done in the Clinical Practice

The evidence on pregnancy and dialysis is heterogeneous and several questions are still unanswered. Two of them are of pivotal importance: when to start dialysis in pregnancy and how we can mediate between the excellent results obtained by Hladunewich in Canada with long-hours daily dialysis and the limited access to dialysis in many developing countries.

The issue of dialysis start in pregnancy is complex; the Canadian data, which indicate that a target of predialysis “near normal” urea level is associated with better outcomes, are often taken as an indication to start dialysis early. While this attitude may be reasonable, it has not been proven, and is not in keeping with the recent indications to start dialysis within an “intent to delay” policy in all other categories of patients [111,119,120,121,122].

As a consequence of this uncertainty, the residual kidney function at dialysis start in pregnancy ranges from 20 mL/min, a level that would not have supported the indication to start RRT outside of pregnancy, to the usual levels of less than 10 mL/min [123,124].

There are very few position statements from scientific societies on these issues; the most recent Italian one leaves the question open, advocating an individualised approach [6,111]. Furthermore, the role of nutritional support to delay dialysis start in pregnancy has been insufficiently studied, and the promising results obtained with plant-based diets need large-scale validation [125,126,127]. There is an urgent need for gathering and exchanging data on this crucial issue to ensure the best timing of start of dialysis in pregnancy, in the interest of the mothers and the babies.

The second issue, regarding the difficulty in implementing the long, daily dialysis schedules, is also open; this is of utmost importance in developing countries, such as Mexico, where intensive dialysis may lead to a competition for a lifesaving treatment [92]. Shorter dialysis schedules and peritoneal dialysis are reported as alternatives, but publication bias is an important limit to assess the real equivalence of these more easily manageable treatments [128,129,130,131,132]

Low income should not become synonymous with low quality, and data coming from medium–low-income countries show that very good results can be obtained in all settings; the populations may, however, be different, as well as the kidney diseases. There is a need for establishing a common language, allowing exchanging on dialysis approaches and detailed results to allow tailoring dialysis to patients, considering also the available resources. Some open questions regarding the management of the dialysis schedules are summarized in Table 5.

## 4. Kidney Transplantation

Fertility is at least partly restored after kidney transplantation, and the possibility to undertake a successful pregnancy is usually considered an added value of a functioning kidney graft. Reports on pregnancies after kidney transplantation rapidly followed the development of kidney transplant programs and thousands of pregnancies have now been reported all over the world [133,134,135,136,137,138,139,140].

However, even in an ideal situation, the risk of complications is higher than in the general population and was recently described as corresponding to stage-1 CKD in native kidneys, in patients with potentially progressive CKD [140,141]. If teratogen drugs are avoided (Appendix Table A1, Table A2, Table A3 and Table A4), pregnancy after kidney transplantation shares the same risk factors with CKD pregnancies, suggesting that kidney function, hypertension, and proteinuria matter more than treatments [141]. Indeed, the reduced nephron mass of a solitary transplanted kidney may not be resilient enough to the stressors of hyperfiltration of pregnancy, even in the presence of normal kidney function.

The profile of the “ideal” candidate for pregnancy after transplantation is well defined: a young, nonobese, normotensive woman, with normal kidney function, in the absence of proteinuria, without any rejection episode, or at least any recent rejection episode, with good compliance, and at least two years from transplantation [133,134,140,141,142,143,144]. However, a univocal definition of age, kidney function, and interval after the last rejection episode is missing, and the grading of the risks is not clear, in particular in women with signs of kidney function impairment. This “ideal” situation is indeed not always the rule, in particular in deceased donor transplantation. In fact, expanded donor policies may lead to suboptimal kidney function; higher age at transplantation and reduced fertility are not infrequent; conversely, the good results of pregnancy after kidney transplantation are somehow smoothing the contraindications, with a widespread agreement on a shorter stabilization time, and a permissive attitude towards pregnancy with less-than-optimal kidney function [133,134,135,136,137,138,139,140,141,142,143,144].

The literature is rich with reports on extreme situations, including kidney transplantation during pregnancy; while single cases cannot lead to potentially risky changes in daily practices, such cases do warn against fully negative attitudes and suggest an individually based decision [145,146,147,148,149,150,151].

In particular in western countries, where age of the transplant recipients is often older, assisted fertilization is increasingly popular. Besides the ethical challenges, whose discussion is beyond the scope of this review, assisted fertilization techniques, in particular those in vitro, are associated with an increase of pregnancy complications and of hypertensive disorders of pregnancy, with a potential negative effect on the kidney function [152,153,154,155,156]. The scattered data on assisted fertilization in kidney transplant recipients are, however, encouraging; once more, the series are very small, most of them regarding single cases, and, in such a context, it is highly probable that reports are influenced by a publication bias [152,153,154,155,156].

### What Is Missing and Could Be Done in the Clinical Practice

Most of the available data on kidney transplantation regard pregnancies in ideal or “almost ideal” clinical conditions; there is very little evidence available on pregnancy in patients with reduced kidney function. In these cases, in which counseling is particularly important, the data we now have are scant and we can rely only on personal experience or indirect evidence.

There is a need for gathering data on these situations, to improve counseling and to increase awareness of the challenges of pregnancy in patients with a failing graft.

Pregnancy represents an immunologic challenge and is a potential cause of hyperimmunization, a well-known problem for transplantation. Living unrelated grafts increasingly offer a clinical solution within couples: the eventual role of pregnancy as an immunologic trigger leading to rejection of the grafted kidney should be clarified to optimize the outcomes of transplantation between husband and wife [142].

Not least, long-term data on children born from a transplanted mother are still lacking, and attention should be focused on this important issue [157]. 

## 5. Conclusions

Pregnancy is now possible in all phases of chronic kidney disease, but its management may be difficult and the outcomes are not the same as in the overall population.

There is a lot to do to improve pregnancy outcomes in CKD, whose prevalence in pregnancy is probably as high as that of PE, a better-acknowledged risk for adverse outcomes. When CKD is known, pregnancy should be considered as at high risk and followed accordingly, since the earliest stages. Furthermore, since CKD is often asymptomatic, pregnant women should be screened for the presence of CKD, allowing better management of pregnancy and timely treatment after pregnancy. The differential diagnosis between CKD and pre-eclampsia may be difficult but is important for pregnancy management.

Women on dialysis should be aware of the possibility of conceiving and having a successful pregnancy, and intensive, long-hours, quotidian dialysis is the choice allowing the best results; these indications have, however, to be adapted to poorly resourced countries, where daily dialysis may not be feasible for clinical, economical, or logistic issues.

After kidney transplantation, pregnancies should be followed up with great attention to minimize the risks for mother, child, and for graft.

Specialized and committed teams are crucial for optimizing the care of pregnant CKD patients, and efforts should be done to organise such teams.

The advances in our knowledge are strictly linked to the progress in research, and international comparisons are highly needed to ameliorate our understanding and to define treatment strategies. The research agenda is long, from epidemiology of CKD to differential diagnosis between CKD and PE, evaluation of target blood pressure in CKD pregnancies, or the role of the nutritional follow-up. Furthermore, dialysis start, dialysis policy, and modulation of posttransplant therapies should be discussed on a global basis, to identify the best context-sensible policies to allow more CKD women to attain successful pregnancy.

## Figures and Tables

**Table 1:** Clinical features affecting pregnancy-related risks in CKD patients.

Clinical Feature	Effect on Pregnancy	Effect on Maternal Health
CKD stage	Increase across stages (from stage 1 to stage 5) of preterm delivery, caesarean section, SGA	Increase in risk of kidney function impairment, development of hypertension, and proteinuria
Immunologic diseases	PE risk may be increased at least in some diseases (IgAGN, SLE); differential diagnosis with flares may be difficult	In CKD, maternal deaths are mainly described in SLE nephropathy (immunologic flares)
Diabetes and diabetic nephropathy	Increased risk in malformations, linked to diabetes, proportional to diabetes control	Same as CKD
Baseline hypertension	May be associated with a higher risk of preterm or SGA babies	Risk of kidney function impairment may be increased
Baseline proteinuria	May be associated with a higher risk of preterm or SGA babies	Risk of kidney function impairment and of persistent hypertension may be increased

CKD: chronic kidney disease; IgAGN: IgA nephropathy; SLE: systemic lupus erythematous; SGA: small for gestational age baby.

**Table 2:** Adverse pregnancy outcomes in CKD patients and in their offspring [13].

Term	Definition	Main Issues in CKD
Maternal death	Death in pregnancy or within 1 week–1 month postpartum	Too rare to be quantified, at least in highly resourced settings, where cases are in the setting of severe flares of immunologic diseases (SLE in primis).
CKD progression	Decrease in GFR, rise in sCr, shift to a higher CKD stage	Differently assessed and estimated; may be linked to obstetric policy (anticipating delivery in the case of worsening of the kidney function); 20% and 80% in advanced CKD. Probably not increased in early CKD (stages 1–3a).
Immunologic flares and neonatal SLE	Flares of immunologic diseases in pregnancy	Risks are mainly but not exclusively limited to patients who start pregnancy with active disease or recent flare-up. Definition of a “safe” zone is not agreed; in quiescent, well-controlled diseases are probably equivalent to nonpregnant, carefully-matched controls.
Transplant rejection (kidney)	Acute rejection in pregnancy	Kidney rejection episodes are not increased with respect to matched controls; may be an issue in unplanned pregnancies.
Abortion	Foetal loss, before 21–24 gestational weeks	Scant data on advanced CKD. An issue in immunologic diseases (not exclusively linked to LLAC) and in diabetic nephropathy.
Stillbirth	Delivery of a nonviable infant, after 21–24 gestational weeks	Probably not increased in early CKD, too few data in late CKD stages; may be an issue in dialysis patients; when not linked to extreme prematurity, may be specifically linked to SLE, immunologic diseases, and diabetic nephropathy.
Perinatal death	Death within 1 week–1 month from delivery	Usually a result of extreme prematurity, which bears a risk of respiratory distress, neonatal sepsis, cerebral haemorrhage.
Small, very small baby	A baby weighing <1500–2500 g at birth	Gestational age adjusted weight may be a better risk marker; however, very low birth weight may bear risks for future health, even without SGA. Different cut-points are used, the most common are 1500 for very small and 2500 for small baby.
Preterm, early extremely preterm	Delivery before 37, 32, or 34; 28 completed gestational weeks	Risk of preterm delivery increases across CKD stages. Definition of preterm and extremely preterm are agreed (<37 and <28 weeks, respectively); two cut-points are used for early preterm (<32 and <34 weeks)
Small for gestational age (SGA)	<5th or <10th centile for gestational age	SGA and IUGR may be associated, but IUGR is also a dynamic event, characterised by flattening of the growth curve. Both are better defined when several data in pregnancy are available.
Intrauterine growth restriction (IUGR)	<5th centile or flattening of the growth curve	Small, SGA, and IUGR babies are at higher risk of developing hypertension, metabolic syndrome, and CKD in adulthood.
Malformations	Any kind of malformations	Malformations are not increased in the absence of teratogen drugs; exception is diabetic nephropathy (increase in malformations attributed to diabetes); hereditary diseases may not be evident at birth.
Hereditary kidney diseases	Any kind of CKD	Several forms of CKD have a hereditary pattern or predisposition, among them, ADPKD, Alport’s, vescico ureteral reflux and CAKUT, IgA, kidney tubular disorders, and mitochondrial diseases.
CKD-hypertension	Higher risk of hypertension and CKD in adulthood	Lower nephron number in preterm babies; the risks are probably higher in SGA–IUGR babies than in preterm babies adequate for gestational age.
Other long-term issues	Developmental disorders	Cerebral haemorrhage or neonatal sepsis, not specific of CKD, are a threat in all preterm babies.

CKD: chronic kidney disease; ADPKD: autosomal dominant polycystic kidney disease; CAKUT: complex anomalies of the kidney and of the urinary tract; SLE: systemic lupus erythematous; LLAC: lupus-like anticoagulant; SGA: small for gestational age baby; IUGR: intrauterine growth restriction; GFR glomerular filtration rate; MMF: mycophenolic acid; ACEi: angiotensin-converting enzyme inhibitors; ARBS: angiotensin receptor blockers.

**Table 3:** The differential diagnosis of disorders associated with AKI and thrombocytopenia in pregnancy (modified from 70).

	Pre-Eclampsia/HELLP	Hemolytic Uremic Syndrome (HUS)	Thrombotic Thrombocytopenic Purpura (TTP)
Usual time of onset	After 20 gestational weeks	postpartum	2nd and 3rd trimester
Arterial hypertension	+++	+	+
Renal failure	+ (mainly in HELLP)	+++	+
Renal prognosis	Recovery	75% ESRD	Usually good
Neurological impairment	+	+	+++
Hemolytic anemia	++ (HELLP)	+++	+++
Low platelet count	+++ (HELLP)	+++	+++
Intravascular coagulation	+	Usually absent	++
Proteinuria	+++	+	Rare
Abnormal liver function tests	+++ (HELLP)	Usually absent	Usually absent
Complement alternative pathway activation	+ (HELLP)	+++	Usually absent
Reduced ADAMTS13	+	Usually absent	+++
Treatment	Supportive treatment	Plasmapheresis; plasma infusion Anti-MAC antibodies	Plasmapheresis; plasma infusion Anti-CD20 antibodies

HELLP: hemolysis, elevated liver enzymes, low platelets. + present; ++ frequent and severe; +++ very frequent and severe.

**Table 4:** Challenges for the diagnosis of kidney diseases in pregnancy.

Sign or Symptom	Interference in Pregnancy	Potential Correction
Hypertension	Blood pressure is physiologically reduced, particularly in the 1st trimester; mild–moderate hypertension may be masked by the physiologic changes in early pregnancy.	Attention to “high normal” BP; strict monitoring in presence of risk factors (obesity, diabetes, family history, age, CKD); if associated with proteinuria, interference in PE diagnosis.
Kidney function reduction	Kidney function physiologically increased, in particular in 1st–2nd trimester; CKD stages 2–3 may be missed, in particular if reference eGFR of nonpregnant women is employed.	Lack of hyperfiltration is a potential sign of initial reduction of kidney function; “normal” values outside pregnancy should be interpreted with caution in pregnancy.
Hematuria	Presence of contaminant RBCs in the urine is common and microhematuria may be misinterpreted as of gynaecologic origin. Possible underestimation in polyuria.	Microscopic examination, search for urinary casts, and attention to correct sampling avoid missing this sign pointing to a glomerular origin (most frequent, IgA nephropathy).
Proteinuria	The physiologic limit is increased to 300 mg/day. Proteinuria may show day-to-day and circadian variations. If assessed on spot urine collection, mild proteinuria may be missed in patients with polyuria.	Repeated tests and 24 h urine assessment in cases with trace proteinuria may avoid missing low-grade (albeit clinically relevant) proteinuria.
Tubular derangements	The usual workout of physiologic pregnancy includes the major ions (Na, K, Calcium). Mild hypokalaemia and hyponatremia are common, due to physiologic hemodilution.	Tubular derangements may be elusive. Phosphate, Mg, bicarbonate should be controlled in severe hypokalaemia, severe hyponatremia, or unexplained polyuria.
Urinary infection	Urinary infections are common and usually asymptomatic. Several societies suggest at least one urinary culture per trimester.	Repeatedly positive urinary cultures suggest performing ultrasounds of the kidney and urinary tract.

CKD: chronic kidney disease; PE: pre-eclampsia; BP: Blood pressure; RBCs: red blood cells. Na: sodium, K: potassium, eGFR: estimated glomerular filtration rate.

**Table 5:** Some challenges and open questions in the practical management of hemodialysis patients in pregnancy.

Item	Indications	Open Issues
Number of sessions	Daily hemodialysis (DHD) is superior to thrice weekly dialysis	DHD is differently defined (5–7 sessions/week); no data on adjustment per body size or residual renal function (RRF): urea level should probably guide the choice. Unclear advantage of shorter/more frequent or of longer/less frequent sessions in presence of RRF.
Duration of session	The longest, the best, according to the Canadian experience	Scattered positive experience with short DHD; difficult to distinguish between effect of frequency and duration. Long-hours dialysis is more effective in removal of middle molecules and phosphate.
Weight loss	The lowest, the best; attention to avoid dehydration and oligoamnios	The evaluation of weight gain remains empiric. Unclear role for bioimpedance, BNP, BP. Unclear role of biomarkers (s-Flt1, PlGF) in diagnosis of PE.
Blood flow	Low blood flow (150–250 mL/min) may be reasonable in intensive HD	Lower blood flow has to be balanced with higher need for anticoagulation. Long-hours dialysis is associated with better removal of middle molecules and compartmentalised toxins: depuration markers alternative to urea are not identified. Hypophosphatemia is often found: subtle deficits of trace metals or vitamins may be relevant; the role of multivitamin supplementation is not clear.
Dialysate flow	Low dialysate flow advised in long-hours dialysis (300 mL/min)	
Dialysis efficiency	The most common target is “near normal” urea (10–15 umol/L) following the day break	
Dialysis membrane	Biocompatible membranes	No study addressed to different membranes; low-flux membranes limit nutrient loss; high-flux membranes increase middle molecules clearance, but increase also nutrient loss and may induce back-filtration.

CKD: chronic kidney disease; PE: pre-eclampsia; BP: blood pressure; BNP: brain natriuretic peptide; HD: haemodialysis; DHD: daily haemodialysis; s-Flt1: soluble fms like tyrosine kinase 1; PlGF: placental growth factor; RRF: residual renal function.

## Appendix A

**Table A1:** Main immunosuppressive drugs for CKD patients in pregnancy.

Drug	Main Features	FDA
**Usually considered as relatively safe, when absolutely needed**		
Azathioprine (AZA)	It is teratogenic in animal models, at high doses, but not in humans, possibly because the foetal liver is not able to activate the drug. KDIGO and European Best Practice Guidelines suggest switch from Mycophenolate to AZA before pregnancy.	D
Cyclosporine A (CyA)	Hypertension, hyperglycaemia and nephrotoxicity may be relevant in pregnancy. CyA has not been associated with teratogenicity; SGA babies and preterm delivery have been reported, with an unclear link with maternal disease; levels vary in pregnancy and need strict monitoring.	C
Tacrolimus	The drug has similar effects and side effects as CyA; the experience is more limited than with CyA.	C
Steroids	The most frequently used short-acting corticosteroids are prednisone, methylprednisolone and prednisolone; among long-acting betamethasone and desamethasone. No major malformation reported, increase of labio-palatoschisis is debated. A risk of premature rupture of membranes has been reported, along with increased risk of infection and gestational diabetes.	C prednisone
Hydroxychloroquine	This synthetic antimalaric agent crosses the placenta but was not associated with foetal toxicity.	B
IV Immunoglobulin (IV Ig)	IV Ig is indicated in SLE pregnant patients with recurrent spontaneous abortion. Safety has not been fully established.	C
Rituximab	Animal studies have shown adverse effects on the fetus; no adequate studies in humans. Benefit may warrant use in highly selected cases.	C
Belimumab	In exceptional cases. No adequate studies in humans. Benefit may warrant use despite potential risks, in highly selected cases.	C
**To be avoided**		
Cyclophosphamide	Reports suggest that pregnancy termination is common in case of inadvertent use. A few positive reports, mainly in SLE are available.	D
Methotrexate	Also employed for extrauterine pregnancy termination. Discontinuation for one–three menstrual cycles pre-conception is usually indicated.	X
Mycophenolate	Severe foetal malformations are reported, mainly cardiovascular and cranial. Discontinuation for at lest 6 months, also for stabilizing the kidney function, is usually indicted after kidney transplantation.	D
m-Tor inhibitors	Very few studies regard their use in pregnancy. They are teratogenic in animals; discontinuation in humans is matter of debate; KDIGO guidelines suggest discontinuation in prevision of pregnancy.	C
Thymoglobulin	Animal studies are not available. There are no controlled data in human pregnancy.	NA
Basiliximab	Animal studies failed to reveal embryotoxicity, or teratogenicity. IgG are cross the placental barrier and the IL-2 receptor plays an important role in development of the immune system. There are no controlled data in human pregnancy.	B
Alemtuzumab	In animal studies no teratogenic effects are observed. However, there was an increase in embryolethality in pregnant animals. There are no controlled data in human pregnancy, but potential benefits may warrant use of the drug in selected pregnant women despite potential risks.	C

FDA site of the Food and Drug Administration; FDA rating: B. No evidence of risk in studies; C. Risk cannot be ruled out; D. Positive evidence of risk; X. Contraindicated in pregnancy. NA. not available.

**Table A2:** Main anti-hypertensive drugs for CKD patients in pregnancy.

Drug	Main Features	FDA
**Usually considered as first choice**		
Alpha-methyldopa	Widely used in pregnancy, with no reported negative effects. May not be sufficient to correct severe hypertension in CKD.	B
Niphedipine	The long acting form is commonly used. In CKD the side effect of increasing peripheral oedema may be relevant.	C
Labetalol	Usually well tolerated, should be avoided in asthma. In a RCT it was comparable to alpha-methyldopa.	C
**Usually considered as second choices**		
Beta-blockers	The main negative effect in older studies was foetal growth restriction, possibly as an effect of overzealous correction of BP. Beta1 selective beta-blockers (atenolol) are more often in cause. May be more effective than alpha-methyldopa, alone or in combined therapy. At delivery they may induce hypoglycaemia, hypotension and bradycardia (usually mild and transient).	D atenolol B pindolol C metoprol
Clonidine	The effect is similar to alpha-methyldopa; side effects may be more common and hypertensive rebounds at discontinuation are common; slowing fetal growth is occasionally reported.	C
Diuretics	They are usually avoided in pregnancy except for cardiological indications. Thiazides may be continued in patients previously on treatment. In Gitelman syndrome, аmiloride may be needed.	B hydrocloro-thiazide
**To be avoided**		
Niphedipine short acting	Contraindicated by FDA, RCOG and AIPE for the risk of severe sudden hypotension with detrimental effect on placental flows.	X
ACE-i/ARBs and related drugs	Both are contraindicated in all phases of pregnancy; different malformations, involging cardiovascular, central nervous system, renal and bone are reported. Recent studies suggest that the risk is limited to the second and third trimester.	X

FDA site of the Food and Drug Administration; FDA rating: B. No evidence of risk in studies; C. Risk cannot be ruled out; D. Positive evidence of risk; X. Contraindicated in pregnancy. RCT: randomized controlled trial. RCOG: Royal College of Obstetricians and Gynecologists. AIPE—Italian Association on Preeclampsia.

**Table A3:** Main antibiotics for CKD patients in pregnancy.

Drug	Main Features	FDA
**Usually considered as first or second choices**		
Ampi-amoxycillin	Ampicillin and Amoxicillin are the first-choice antibiotics.	B
Clavulanic acid	Indicated when therapy with the previous ones is not effective, or according to antibiogramme.	B
Cephalosporins	Available data do not indicate an increase of malformations; risk for kernicterus is increased (mainly ceftriaxone).	B
Carbapenems	Meropenem is the first choice in severe infection; no demonstrated risks in humans; increased risk of malformations with imipenem-cilastatin in animals.	B
Aztreonam	Alternative in case of allergy to beta-lactams (parenteral only).	B
Macrolides	Eritromicine is a good alternative in case of contraindication to beta-lactamics. Claritromicine and azitromicine are a second choice.	B
Phosphomycin	Indicated in uncomplicated urinary tract-infections.	B
Nitrofurantoin	Contraindicated in G6PDH-deficiency. Contraindicated at the end of the pregnancy for risk of haemolytic anaemia in the new-born.	B
**To be avoided (except when lifesaving)**		
Aminoglycosides	Associated with ototoxicity in the foetus and newborn.	D
Fluoroquinolones	Associated with abnormalities in the development of cartilages in animal studies	C
Tetracycline	Cause of various bone abnormalities.	D
Sulphonamides	Sulfamethoxazole/trimethoprim is a folic acid antagonist that increases the risk of kernicterus.	D

FDA site of the Food and Drug Administration; FDA rating: B. No evidence of risk in studies; C. Risk cannot be ruled out; D. Positive evidence of risk.

**Table A4:** Other commonly prescribed drugs for CKD patients in pregnancy.

Drug	Main Features	FDA
**Usually considered as relatively safe, when absolutely needed**		
Acetylsalicylate	At low doses may protect against pre-eclampsia; discontinuation before delivery is recommended.	A
Low molecular weight heparin	Do not cross the placenta. Safe for prophylaxis and treatment of thromboembolic complications in pregnancy and post-partum.	B
ESAs	Do not cross the placenta and are considered safe; there may a need to increase doses in pregnancy.	Not assigned
Allopurinol	Crosses the placenta. Animal reproduction studies have shown potential adverse effects on the fetus. No adequate studies in humans. Potential benefits may warrant use of in pregnant women despite potential risks.	C
Vitamin D	No advantage when given regardless of blood levels Vitamin D3 is recommended to correct deficiency. Cholecalciferol crosses the placenta but the transfer to the fetus is low; no evidence of adverse effects. Calcitriol, paricalcitol are teratogenic in animal studies. Animal studies have shown adverse effects of Ergocalciferol on the fetus. No adequate study in humans.	Not assigned Cholecalciferol C Ergocalciferol
Iron supplements	Multivitamin with iron is only recommended for use in pregnancy when benefit outweighs risk. Ferrous sulfate use is the most frequently used. Maternal anemia increases the risk of low birth-weight, premature delivery, and impaired cognitive development. Recent studies have linked high serum iron with an increased risk of gestational diabetes	A Multivitamin with iron Not assigned Ferrous sulfate
Sodium bicarbonate	No animal or human data availale. Sodium bicarbonate should only be given during pregnancy when benefit outweighs risk	C
Calcium carbonate	Malformation risk is unlikely in humans. In some patients, permanent hypercalcemia resulted in adverse effects on the fetus. No available data in humans. Low intake is associated with adverse pregnancy events.	Not assigned
**To be avoided**		
Warfarin	Evidence of teratogenicity in animal studies. In humans, exposure during the first trimester caused congenital malformations in about 5% of the exposed. Mental retardation, blindness, schizencephaly, microcephaly, hydrocephalus, and other adverse pregnancy outcomes have been reported following exposure in the second and third trimesters. Fatal fetal hemorrhage and an increased risk of spontaneous abortion and fetal mortality are also reported. Case by case evaluation in the case of cardiac indications.	Not assigned
Novel anticoagulants	Animal studies show adverse effects for Rivaroxaban, Dabigatran and Edoxaban. No adequate study in humans, but potential benefits may warrant use in highly selected cases.	C
Calcimimetics	Animal studies show adverse effects, and there is no adequate study in humans; potential benefits may warrant use in highly selected cases.	C
Sevelamer	Animal studies show adverse effects, and there is no adequate study in humans; potential benefits may warrant use in highly selected cases	C

FDA site of the Food and Drug Administration; FDA rating: A. Controlled human studies show no risk; B. No evidence of risk in studies; C. Risk cannot be ruled out.
